# The Aseptic Treatment of Wounds

**Published:** 1895-09

**Authors:** 


*The Aseptic Treatment of Wounds.* By DR. C. SCHIMMELBUSCH. Translated from the Second German Edition by ALFRED THEODORE RAKE, M.B., B.S. Pp. xvi., 250. London: H. K. LEWIS. 1894.

This little book is an exposition of the method of wound-treatment adopted in Von Bergmann's Clinic—the method, that is, of aiming at asepsis by a process of sterilisation rather than by the action of antiseptics. The method is clearly and fully described, and Dr. Schimmelbusch has been at much pains in endeavouring to substantiate his position by bacteriological evidence, which leads him to conclusions very diverse from those of many eminent authorities. He prefaces his description of the details of the method by a useful little chapter on the germs of wound infection, and a more elaborate one on the means of disinfection; and it is here he appears to confuse the issues, and to be led astray by laboratory experiments. Furthermore, his comparative criticisms of the Listerian method are not of much value, as they show too imperfect an acquaintance with its general working, as well as a complete ignorance of the recent improvements in its *technique*. This controversial element apart, however, the book can be safely recommended as a clear and practical guide to the system in question.

Even in this emancipated field antiseptics cannot, apparently, always be dispensed with; but of these iodoform is the only one that commends itself to our author. The amount of "aseptic fever" expected as the normal and usual result of the majority of graver operations—often between 102° F. and 104° F., and lasting two or three days—will be a surprise to many surgeons; as also, the statement in another chapter that abscesses are "not uncommon" as the result of subcutaneous injections, a calamity that the author attributes to the inefficiency of such substances as carbolic acid for disinfection and the presence of germs in the injected fluids.

A chapter is devoted to the requirements of the operating and sick rooms, and another to the description in detail of a major operation, both which witness to one of the weakest points in this system of asepsis—viz., the absence of all protection against stray germs of infection that may be left or subsequently intrude — an objection whose weight is not lessened by the authoritative statements of its exponents, that "the exclusion of absolutely all germs at an operation, into which so many unavoidable complications enter, can hardly be reckoned on with anything like certainty," and that "the surgeon who does not most carefully stop all bleeding, will reckon in vain on the results and consequences of his antisepsis." The book ends with a very short chapter on the treatment of emergencies, especially in warfare; but we trust that the advice there given for the treatment of compound fractures with small skin wounds of "refraining from slitting up, draining, &c.," and the application of a "simple sealed dressing," is not intended to apply to the ordinary routine of civil practice,—a point which is not made quite clear.

Mr. Rake is to be congratulated on his part of the work, and his translation, written in a simple, easy style, has laid English readers under a real obligation to him. An ample bibliography is appended.

